# Annotations

**Published:** 1908-04-25

**Authors:** 


### ANNOTATIONS.

### Beneficent Bacteria.

The popular lecture on "Beneficent Bacteria," by Dr. John W. H. Eyre, Bacteriologist to Guy's Hospital, delivered recently at the Institute of Hygiene, was, as its title suggests, a review of the part played by bacterial life in everyday industrial and agricultural processes. The public is becoming daily more familiar with the real and supposed influence of bacteria in the causation of disease, and it is only right that equal publicity should be given to the less sensational and more utilitarian aspects of bacteriology. It seems unjust that virulent micro-organisms alone should attract the attention of the populace, and that the countless millions of really useful bacteria should labour for the benefit of mankind in undeserved obscurity. Dr. Eyre's lecture will therefore serve a double purpose—the education of the public in an instructive sidepath of science, and the vindication of a hardworking and respectable section of the lower vegetable kingdom. Cheese-making, tobacco-curing, tanning, and indigo manufacture, and some of the other more important fields of bacterial utility, were, of course, mentioned; but the lecturer rightly laid particular emphasis on the influence of micro-organisms in the disintegration of dead organic matter. The most useful of our bacterial allies are indeed those which act as universal scavengers. As for the lactic acid bacillus, whom Dr. Eyre mentioned in connection with Metchnikoff's advocacy of sour milk as a beverage, we cannot believe that many people will be found to prefer longevity fostered by a diet of curdled milk, to the usual life-span maintained by the usual methods.

### The Infants Hospital.

Amongst the guiding principles adopted by the authorities of the new Infants Hospital in Vincent Square are some which are distinctly novel. Not all of these may commend themselves to everyone, but others must undoubtedly make for good. Perhaps the most important is that the managers have decided to exclude in future all cases of congenital defect, and we infer from a succeeding paragraph in a lecture by the senior physician that prematurity is held to be such a disqualification, though it is not clear where the line is to be drawn. Perhaps, too, the fact that the staff is composed of physicians only may have had something to do with this decision, for which, so it seems to us, there is a good deal to be said from a practical point of view under present circumstances. It is well known that the checking of our preposterous infant mortality is a problem which for various reasons can be attacked but piecemeal and gradually, and it is therefore strategically sound to concentrate that attack on those points in the position where victory will be best repaid. This is, it is hardly necessary to say, very far from advocacy of an entire refusal of the amelioration of congenital defects or of the upbringing of premature children; although on strictly biological reasoning a case can doubtless be made out even for that, and it is a principle followed out with benefit at the present day by most of the uncivilised peoples in the world. Such efforts no one would wish to see abandoned, except possibly the most advanced school of the disciples of the eugenic faith. But so long as children born healthy and of potential capacity of the very highest utility to the State are undergoing suffering, disease and death in large numbers from preventable causes, it needs no argument to justify their preferential treatment, in hospitals such as this, over the unfortunate victims of interrupted gestation or congenital defects.

### Slaughter House Reform.

The Royal Commission on Vivisection has now concluded its public sittings, and is engaged in the preparation of its report. In the necessary interval that must elapse before the conclusions of the Commission are made public, active anti-vivisectionists may find themselves at a loss for something profitable to do. Decency, of course, forbids anything very violent in the way of meetings or demonstrations, whilst the matter is still *sub judice*. We therefore venture to suggest to these reformers, as a suitable topic for consideration and discussion during their time of inactivity, the humane slaughtering of animals, which was briefly referred to in our issue of last week. This is a subject which should interest all who acknowledge kindly feelings towards the brute creation, and it should especially appeal to those who have embarked upon the campaign against all forms of cruelty to animals for utilitarian purposes. Whole-hearted and consistent humanitarians, who, out of their regard for the sacredness of animal life, abstain altogether from flesh food and condemn the whole craft of the butcher, will protest that the humane slaughtering of animals is a contradiction in terms. To them the recent circular of the Local Government Board, dealing with this matter, will commend itself merely as a palliative measure directed against the more outstanding evils of a practice which is essentially abominable. But all anti-vivisectionists are not vegetarians, and there must be many amongst them who will agree with us that their time of waiting would be well spent in the support of the Admiralty Committee's recommendations for reform in animal slaughter, which have now been circularised by the Local Government Board. The official suggestions are that all animals must be rendered unconscious before blood is drawn; that animals awaiting slaughter must be kept away from the sights and smells of the slaughter-house; that animals slaughtered at the same time in the same chamber must not be in view of each other; and, finally, that slaughtering must be conducted in privacy by licensed and qualified slaughterers only. Should these recommendations become enforced throughout the country, the substitution of public *abattoirs* for private slaughter-houses must almost inevitably follow.

